# Development and Clinical Applications of a 5-Plex Real-Time RT-PCR for Swine Enteric Coronaviruses

**DOI:** 10.3390/v14071536

**Published:** 2022-07-14

**Authors:** Jin-Hui Zhu, Gaurav Rawal, Ethan Aljets, Wannarat Yim-Im, Yong-Le Yang, Yao-Wei Huang, Karen Krueger, Phillip Gauger, Rodger Main, Jianqiang Zhang

**Affiliations:** 1 Department of Veterinary Diagnostic and Production Animal Medicine, Iowa State University, Ames, IA 50011, USA; miazjh@iastate.edu (J.-H.Z.); grawal@iastate.edu (G.R.); ealjets@iastate.edu (E.A.); wyimim@iastate.edu (W.Y.-I.); kharmon@iastate.edu (K.K.); pcgauger@iastate.edu (P.G.); rmain@iastate.edu (R.M.); 2 Department of Veterinary Medicine, Zhejiang University, Hangzhou 310058, China; yang-yl@zju.edu.cn (Y.-L.Y.); yhuang@zju.edu.cn (Y.-W.H.)

**Keywords:** swine enteric coronavirus, PEDV, PDCoV, TGEV, SADS-CoV, 5-plex real-time RT-PCR

## Abstract

A PEDV/PDCoV/TGEV/SADS-CoV/XIPC 5-plex real-time RT-PCR was developed and validated for the simultaneous detection and differentiation of four swine enteric coronaviruses (PEDV, PDCoV, TGEV and SADS-CoV) in one PCR reaction (XIPC serves as an exogenous internal positive control). The 5-plex PCR had excellent analytical specificity, analytical sensitivity, and repeatability based on the testing of various viral and bacterial pathogens, serial dilutions of virus isolates, and in vitro transcribed RNAs. The 5-plex PCR had comparable diagnostic performance to a commercial PEDV/TGEV/PDCoV reference PCR, based on the testing of 219 clinical samples. Subsequently, 1807 clinical samples collected from various U.S. states during 2019–2021 were tested by the 5-plex PCR to investigate the presence of SADS-CoV in U.S. swine and the frequency of detecting swine enteric CoVs. All 1807 samples tested negative for SADS-CoV. Among the samples positive for swine enteric CoVs, there was a low frequency of detecting TGEV, an intermediate frequency of detecting PDCoV, and a high frequency of detecting PEDV. Although there is no evidence of SADS-CoV presence in the U.S. at present, the availability of the 5-plex PCR will enable us to conduct ongoing surveillance to detect and differentiate these viruses in swine samples and other host species samples as some of these coronaviruses can cause cross-species infection.

## 1. Introduction

Coronaviruses (CoVs) are a group of highly diverse, enveloped, positive-sense and single-stranded RNA viruses in the order *Nidovirales* and the family *Coronaviridae*. Four genera, *Alphacoronavirus*, *Betacoronavirus*, *Gammacoronavirus*, and *Deltacoronavirus*, have been described for CoVs. In pigs, six CoVs have been identified. These include porcine epidemic diarrhea virus (PEDV), transmissible gastroenteritis virus (TGEV), porcine respiratory coronavirus (PRCV), and swine acute diarrhea syndrome coronavirus (SADS-CoV) belonging to the alphacoronaviruses, porcine hemagglutinating encephalomyelitis virus (PHEV) belonging to the betacoronaviruses, and porcine deltacoronavirus (PDCoV) belonging to the deltacoronaviruses [1]. PEDV, TGEV, PDCoV, and SADS-CoV primarily cause enteric infections in pigs. PRCV is a mutant of TGEV with a large deletion in the spike gene and, in contrast to TGEV that mainly targets the enteric tract, PRCV has a predilection for the respiratory tract [1]. PHEV infection (“vomiting and wasting disease”) produces encephalomyelitis rather than enteritis, and thus is not often considered when differentiating enteric infections. Therefore, the four coronaviruses (PEDV, TGEV, PDCoV, and SADS-CoV) that cause indistinguishable clinical signs, such as loss of appetite, diarrhea and vomiting, weight loss, lethargy, and death [2], are often considered as swine enteric coronaviruses and differential diagnosis of them is needed.

TGEV was first described in 1946 in the U.S. [3] and subsequently reported worldwide. Nowadays, TGEV is still endemic in the U.S., although the prevalence is low [4]. PEDV was first reported in England in the 1970s and it subsequently spread across most of Europe, followed by detection in some Asian countries in the 1980s and thereafter [5,6,7]. The emergence of highly virulent PEDV strain associated with high mortality of suckling piglets in China in late 2010 [8,9] attracted great attention and concern. A highly virulent PEDV strain was detected for the first time in North America in 2013 [10] and this strain was referred to as the “U.S. original PEDV strain”, “U.S. prototype PEDV strain”, or “non-S INDEL strain” [11,12,13,14]. A clinically milder PEDV variant strain was identified in 2014 in the U.S. [15] and it was called “S-INDEL strain” [13]. Subsequently, PEDV emerged or re-emerged in multiple Asian and European countries [16]. PEDV remains a significant challenge to the global swine industries. PDCoV was first reported in the feces of domestic pigs in Hong Kong, China in 2012 [17] but its association with pig diarrhea was not reported until 2014 in the U.S. and the virus rapidly spread to multiple states in the U.S. [18,19]. Subsequent to the U.S. introduction, PDCoV has since been detected in many other countries such as Canada, mainland China, Thailand, South Korea, Japan, Lao People’s Democratic Republic, Vietnam, Mexico, and Peru [20,21,22,23]. SADS-CoV was first detected in China in 2017 [24,25,26] and, in addition to the name SADS-CoV, other names such as porcine enteric alphacoronavirus (PEAV) and swine enteric alphacoronavirus (SeACoV) have also been used to describe this virus [24,25]. So far, SADS-CoV has only been reported in two provinces (Guangdong and Fujian) in China [27] and it remained unknown whether or not it was present in U.S. swine.

Since the swine enteric CoVs cause similar clinical signs, lab-based differential diagnosis is necessary. Reverse transcription real-time PCR (RT-rtPCR) is a sensitive and specific assay and has been widely used for detection of many RNA viruses, including swine enteric coronaviruses. For example, the TaqMan probe-based singleplex RT-rtPCR for PEDV [28,29], PDCoV [30,31,32,33], TGEV [34], and SADS-CoV [35,36,37]; the duplex RT-rtPCR for PEDV and PDCoV [38]; the duplex RT-rtPCR for PEDV and TGEV [39]; the triplex RT-rtPCR for PEDV, PDCoV and TGEV [40]; and the triplex RT-rtPCR for PEDV, PDCoV and SADS-CoV [41] have been reported. Recently, a PEDV, PDCoV, TGEV, and SADS-CoV multiplex RT-rtPCR was reported by a group in China [42]. However, there are concerns with some previously described PCR assays. For example, in the PEDV/TGEV duplex PCR [39] and in the PEDV/PDCoV/TGEV/SADS-CoV multiplex PCR [42], the TGEV PCR primers and probes targeting the nucleocapsid gene reacted with both TGEV and PRCV; in addition, neither of these two PCRs contained an internal positive control. In 2018, novel sparrow deltacoronaviruses were identified [31] and it was then found that the previously developed membrane gene-based PDCoV PCR [30] and nucleocapsid gene-based PDCoV PCR [31] cross-reacted with sparrow deltacoronaviruses.

In this study, we developed and/or evaluated multiple singleplex RT-rtPCR assays for each swine enteric CoV and then selected one singleplex RT-rtPCR each for the detection of PEDV, PDCoV, TGEV, and SADS-CoV, together with a singleplex PCR for an exogenous internal positive control (XIPC) to develop the PEDV/PDCoV/TGEV/SADS-CoV/XIPC 5-plex RT-rtPCR (simplified as 5-plex PCR thereafter in this paper). After thorough validation, the 5-plex PCR was used to screen a large number of clinical samples collected from U.S. swine during 2019–2021 to investigate whether SADS-CoV is present in the U.S. and also to determine the detection frequency of swine enteric CoVs in U.S. swine.

## 2. Materials and Methods

### 2.1. Primers and Probes of PCRs Included in This Study

The sequences of PCR primers and probes included in this study are shown in Table 1. Two previously published TaqMan probe-based singleplex assays, SADS-CoV PCR assay 1 [35] and assay 2 [36], and two TaqMan probe-based singleplex SADS-CoV PCR assays developed in the current study (assay 3 and assay 4) were evaluated. In order to select the appropriate singleplex PDCoV, TGEV, and PEDV PCRs for inclusion to develop the 5-plex PCR, two previously published singleplex PDCoV PCRs, i.e., assay 1 [30] and assay 2 [31], and one singleplex PDCoV PCR, i.e., assay 3, developed in this study, were evaluated. Similarly, one published TGEV nucleocapsid (N) gene-based PCR, i.e., assay 1 [42], one published PEDV N gene-based PCR, i.e., assay 1 [28], and one TGEV spike (S) gene-based PCR, i.e., assay 2, and one PEDV N gene-based PCR, i.e., assay 2, developed in this study, were included for evaluation.

Eventually, one singleplex PCR each for PEDV, PDCoV, TGEV, and SADS-CoV together with one XIPC singleplex PCR were selected to develop the PEDV/PDCoV/TGEV/SADS-CoV/XIPC 5-plex PCR with the information pertaining to the primers and probes provided in Table 1. XIPC DNA is a fragment of nucleotides that was artificially designed and synthesized with T7 promoter at the 5′ upstream. The XIPC sequence is not present in any analyzed pathogens or host species. XIPC DNA is in vitro transcribed into XIPC RNA which is added to the extraction lysis buffer before nucleic acid extraction. In the 5-plex PCR master mix, primers and probes specific to XIPC RNA are included. Primers and probes for XIPC were proprietary products developed in our laboratory and their sequences are available upon request.

A commercial PEDV/PDCoV/TGEV PCR, including the internal positive control Xeno (Thermo Fisher Scientific, Carlsbad, CA, USA) was included in this study as a reference PCR for evaluating the PEDV/PDCoV/TGEV/SADS-CoV/XIPC 5-plex PCR. The information of the primers and probes of this commercial PCR is unavailable to us.

### 2.2. Viral and Bacterial Pathogens

One PEDV non-S INDEL isolate USA/IN19338/2013 [43], one TGEV Purdue strain (ATCC VR-763), one TGEV Miller strain (ATCC VR-1740), and one PDCoV isolate USA/IL/2014 [30] were included in this study for assay evaluation. These isolates are available at the Iowa State University Veterinary Diagnostic Laboratory (ISU VDL). One SADS-CoV isolate GD-01/2017 [44], cultured in Vero cells available from Huang’s laboratory at Zhejiang University, China, was used to validate the singleplex SADS-CoV PCRs and the work was conducted in Huang’s laboratory. One PRCV isolate ISU 1998, one PHEV isolate (NVSL 001-PDV), and two sparrow deltacoronavirus fecal samples (17-690-7 and 17-42824) [31] available at the ISU VDL were included for assay evaluation in this study.

Other viral and bacterial pathogens included in this study for evaluating assay specificity include porcine rotaviruses A, B, and C, porcine circovirus 2, swine influenza A virus, porcine parainfluenza virus 1, pseudorabies virus, Senecavirus A, PRRSV-1 (Type 1), PRRSV-2 (Type 2), some enteric bacterial pathogens (such as *E. coli*, *Salmonella typhimurium*, *Clostridium difficile*, *Clostridium perfringens*, *Brachyspira hyodysenteriae*), and some respiratory mycoplasmas or bacteria (such as *Mycoplasma hyopneumoniae*, *Mycoplasma hyorhinis*, *Mycoplasma hyosynoviae*, *Actinobacillus pleuropneumonia*, *Actinobacillus suis*, *Streptococcus suis*, *Glaesserella (Haemophilus) parasuis*, *Bordetella bronchiseptica*, *Pasteurella multocida*, and *Trueperella pyogenes*). All these pathogens are available at the ISU VDL.

### 2.3. Clinical Samples

For evaluating the diagnostic performance of the singleplex SADS-CoV PCRs, 140 fecal samples collected from Guangdong, Zhejiang, Jiangsu, Shandong, Henan, Hunan, Hebei, Jiangxi, and Anhui provinces, China from 2017–2019 were used. The testing was conducted in Huang’s laboratory in China.

For evaluating the diagnostic performance of the 5-plex PCR in comparison with the reference PEDV/PDCoV/TGEV PCR, 219 clinical samples (54 fecal swabs, 53 feces, 82 oral fluids, and 30 small intestines) collected from various U.S. states and submitted to ISU VDL between 2018 and 2021 were used.

In order to investigate if SADS-CoV is present in U.S. swine, two batches of clinical samples were tested. First, 288 clinical samples, archived during 2019–2020 at the ISU VDL from diarrheic pigs in the U.S. that were negative for PEDV, PDCoV, TGEV, and porcine rotavirus A, B, and C, were tested by the 5-plex PCR. Next, the feces, fecal swabs, and oral fluid samples submitted to the ISU VDL during March-October 2019, November-December 2020, and February–April 2021 were randomly selected for testing by the 5-plex PCR. For the 1519 samples that were positive for at least one of the swine enteric coronaviruses, the detection frequency of single or co-infection of swine enteric coronaviruses was calculated.

### 2.4. Nucleic Acid Extraction

Nucleic acids were extracted from various viral and bacterial pathogens and clinical samples using a MagMAX^TM^ Pathogen RNA/DNA kit (Thermo Fisher Scientific, Waltham, MA, USA) and a Kingfisher Flex instrument (Thermo Fisher Scientific), following the instructions of the manufacturer. The quantity of 100 microliters of the sample was used for extraction, and the nucleic acids were eluted into 90 µL of elution buffer. Before nucleic acid extraction, an internal positive control XIPC RNA (1 × 10^4^ copies per extraction) was added to the extraction lysis buffer. Thus, the extracted nucleic acid from each sample should contain XIPC RNA in addition to the target pathogen nucleic acid.

### 2.5. In Vitro Transcribed RNA

To prepare the RNA standards for SADS-CoV, PEDV, PDCoV, and TGEV, double-stranded and linear gBlock DNA fragments containing the respective CoV genomic region with a T7 promoter at the upstream region was synthesized (Integrated DNA Technologies, Coralville, IA, USA). Specifically, the SADS-CoV gBlock DNA fragment (1165 nucleotides in length) contained the partial SADS-CoV nucleocapsid gene, the PEDV gBlock DNA fragment (1141 nucleotides in length) contained the partial PEDV nucleocapsid gene, the PDCoV gBlock DNA fragment (1066 nucleotides in length) contained the partial PDCoV nucleocapsid gene, and the TGEV gBlock DNA fragment (1138 nucleotides in length) contained the partial TGEV spike gene.

The gBlock DNA fragment was subjected to run-off in vitro transcription into RNA using a MEGAscript T7 Transcription kit (Thermo Fisher Scientific) following the manufacturer’s instructions. RNA transcripts were produced, treated with DNase I and purified by the MEGAclear™ Transcription Clean-Up kit (Thermo Fisher Scientific), following the manufacturer’s instructions. Copy numbers of RNA transcripts were calculated based on concentrations determined by a Nanodrop 2000 spectrophotometer (Thermo Fisher Scientific). Serial dilutions of in vitro transcribed (IVT) RNAs were prepared using a nucleic acid dilution solution (Thermo Fisher Scientific). Aliquots were frozen at −80 °C for single use of each aliquot.

### 2.6. Limit of Detection of Singleplex SADS-CoV PCRs and the PEDV/PDCoV/TGEV/SADS-CoV/XIPC 5-Plex PCR

Serial dilutions of SADS-CoV IVT RNA were tested by singleplex SADS-CoV PCRs to compare their analytical sensitivity. Serial dilutions of IVT RNAs of PEDV, PDCoV, TGEV, and SADS-CoV were tested by the 5-plex PCR with 3 replicates at high concentrations and 20 replicates at low concentrations for each dilution. Then, the limit of detection (LOD) of the 5-plex PCR assay for each virus was determined.

### 2.7. Repeatability of the PEDV/PDCoV/TGEV/SADS-CoV/XIPC 5-Plex PCR

To assess the repeatability of the 5-plex PCR, each dilution of the PEDV, PDCoV, TGEV, and SADS-CoV IVT RNA was tested in triplicate in the same plate for intra-assay repeatability and each dilution of the IVT RNA was tested in three different PCR plates for inter-assay repeatability.

### 2.8. Singleplex SADS-CoV, PDCoV, TGEV, and PEDV PCRs

For all the singleplex SADS-CoV PCR assays 1–4, PDCoV PCR assays 1–3, TGEV PCR assays 1–2, and PEDV PCR assays 1–2, the following procedures were used: Briefly, each PCR was set up in a 20 µL reaction: 5 µL of TaqPath^TM^ 1-Step Multiplex Master Mix No ROX (Thermo Fisher Scientific), 0.3 µL of CoV forward primer at 20 µM, 0.3 µL of CoV reverse primer at 20 µM, 0.25 µL of CoV probe at 20 µM, 0.2 µL XIPC forward primer at 20 µM, 0.2 µL of XIPC reverse primer at 20 µM, 0.25 µL of XIPC probe at 20 µM, 5.5 µL nuclease-free water, and 8 µL nucleic acid extract. Amplification reactions were performed on either an ABI 7500 Fast instrument (Thermo Fisher Scientific) or a QuantStudio 5 instrument (Thermo Fisher Scientific) with the following conditions: 1 cycle of 25 °C for 2 min, 1 cycle of 53 °C for 10 min, 1 cycle of 95 °C for 2 min, and 40 cycles of 95 °C for 3 sec and 60 °C for 30 s. The analysis was undertaken using an automatic baseline, the respective CoV detector at the threshold 0.1, and XIPC detector (Cy5) at 10% of the maximum height of the sigmoid amplification curve.

### 2.9. Reference (Commercial) PEDV/TGEV/PDCoV PCR

A commercial VetMAX^TM^ PEDV/TGEV/PDCoV PCR including primers and probe for Xeno internal positive control (Thermo Fisher Scientific) was included in the present study as a reference PCR (simplified as the reference PCR in this paper). Briefly, 6.50 µL of TaqMan^TM^ Fast 1-Step Master Mix (Thermo Fisher Scientific), 0.80 µL of Amplitaq 360 DNA Polymerase (5 U/µL, Thermo Fisher Scientific), 1 µL of VetMAX PEDV/TGEV/PDCoV Primer Probe Mix (Thermo Fisher Scientific), 3.7 µL nuclease-free water, and 8 µL nucleic acid extract were included in a 20 µL reaction. Amplification reactions were performed on an ABI 7500 Fast instrument (Thermo Fisher Scientific) with the following conditions: one cycle of 50 °C for 5 min, one cycle of 95 °C for 10 min, and 40 cycles of 95 °C for 3 s and 60 °C for 30 s. The analysis was undertaken using an automatic baseline, PEDV detector (LIZ equivalent to Cy5) at the threshold of 5%, TGEV detector (FAM) at the threshold of 5%, PDCoV detector (VIC) at the threshold of 5%, and Xeno detector (NED) at the threshold of 10% of the sigmoid amplification curve’s maximum height, respectively. A threshold cycle (Ct) < 36 was considered positive and a Ct ≥ 36 was considered negative for PEDV, TGEV, and PDCoV.

### 2.10. PEDV/PDCoV/TGEV/SADS-CoV/XIPC 5-Plex PCR

PEDV/PDCoV/TGEV/SADS-CoV/XIPC 5-plex PCR was set up using TaqPath^TM^ 1-Step Multiplex Master Mix (No ROX; Thermo Fisher Scientific). The probes for PEDV, PDCoV, TGEV, SADS-CoV, and XIPC were labeled with the fluorescence dyes JUN, ABY, VIC, FAM, and Cy5, respectively (Table 1).

The concentrations of each virus primers in the 5-plex PCR were optimized at 150 nM, 300 nM, and 900 nM and compared to the corresponding virus singleplex PCR using serially diluted virus isolates and/or IVT RNA. Eventually, the optimized 5-plex PCR was set up in a 20 µL reaction including the following components:

(1). 5 µL of TaqPath^TM^ 1-Step Master Mix (No ROX);
(2). 0.9 µL of PEDV forward primer at 20 µM (final concentration 900 nM), 0.9 µL of PEDV reverse primer at 20 µM (final concentration 900 nM), and 0.25 µL of PEDV probe at 20 µM (final concentration 250 nM);
(3). 0.15 µL of PDCoV forward primer at 20 µM (final concentration 150 nM), 0.15 µL of PDCoV reverse primer at 20 µM (final concentration 150 nM), and 0.25 µL of PDCoV probe at 20 µM (final concentration 250 nM);
(4). 0.3 µL of TGEV forward primer at 20 µM (final concentration 300 nM), 0.3 µL of TGEV reverse primer at 20 µM (final concentration 300 nM), and 0.25 µL of TGEV probe at 20 µM (final concentration 250 nM);
(5). 0.3 µL of SADS-CoV forward primer at 20 µM (final concentration 300 nM), 0.3 µL of SADS-CoV reverse primer at 20 µM (final concentration 300 nM), and 0.25 µL of SADS-CoV probe at 20 µM (final concentration 250 nM);
(6). 0.15 µL of XIPC forward primer at 20 µM (final concentration 150 nM), 0.15 µL of XIPC reverse primer at 20 µM (final concentration 150 nM), and 0.25 µL of XIPC probe at 20 µM (final concentration 250 nM);
(7). 2.15 µL nuclease-free water;
(8). 8 µL nucleic acid extract.

Amplification reactions were performed on a QuantStudio 5 instrument (Thermo Fisher Scientific) with the following conditions: 1 cycle of 25 °C for 2 min, 1 cycle of 53 °C for 10 min, 1 cycle of 95 °C for 2 min, and 40 cycles of 95 °C for 3 s and 60 °C for 30 s. The analysis was undertaken using the Design & Analysis Software 2.6.0 (Thermo Fisher Scientific) with an automatic baseline, PEDV detector (JUN) at the threshold of 4%, PDCoV detector (ABY) at the threshold of 10%, TGEV detector (VIC) at the threshold of 5%, SADS-CoV detector (FAM) at the threshold of 5%, and XIPC detector (Cy5) at the threshold of 10% of the sigmoid amplification curve’s maximum height, respectively.

## 3. Results

### 3.1. Development and Validation of SADS-CoV Singleplex RT-rtPCR Assays

The four SADS-CoV singleplex PCR assays 1–4 specifically reacted with SADS-CoV IVT RNA and did not cross react with other coronaviruses (PEDV, TGEV, PDCoV, PRCV, PHEV, sparrow deltacoronavirus 17-690-7, sparrow deltacoronavirus 17-42824), or other 23 swine viral and bacterial pathogens (Table 2), indicating that all four evaluated SADS-CoV singleplex PCR assays had excellent analytical specificity.

Subsequently, the four SADS-CoV PCR assays were tested using serial dilutions of SADS-CoV IVT RNA. The SADS-CoV PCR assay 1 had values approximately 2 Ct higher than assays 2, 3, and 4 at each IVT RNA dilution, suggesting that the assay 1 was less sensitive than the assays 2, 3, and 4. The SADS-CoV PCR assays 2, 3, and 4 had comparable analytical sensitivity. However, for unknown reason(s), the internal positive control XIPC failed in some reactions of the SADS-CoV PCR assay 1 and assay 4 (data not shown). Hence, the SADS-CoV PCR assay 2 and assay 3 were selected for further evaluations.

When serial dilutions of a SADS-CoV cell culture isolate were tested by SADS-CoV singleplex PCR assays 2 and 3, the two assays had similar detection endpoints at 10^−7^ dilution (Table 3). Although two replicates of the 10^−8^ dilution gave Ct values of 37.2 and 37.1 by the SADS-CoV PCR assay 3, the trend of Ct change did not make sense when compared to that of the 10^−7^ dilution. Thus, the detection endpoint of the SADS-CoV PCR assay 3 was considered as 10^−7^ dilution of the isolate. The SADS-CoV singleplex PCR assays 2 and 3 were further validated using 140 swine fecal samples collected from nine provinces in China. Among the 140 clinical samples, 16 samples were positive by both SADS-CoV singleplex PCR assays 2 and 3, with comparable Ct values for each sample between the two assays, although assay 3 gave slightly lower Ct values compared to that of the assay 2 (data not shown). With all data being considered, the SADS-CoV singleplex PCR assay 3 was selected for developing the PEDV/PDCoV/TGEV/SADS-CoV/XIPC 5-plex PCR. 

### 3.2. Evaluation of PDCoV, TGEV, and PEDV Singleplex PCR Assays

As shown in Table 2, we also evaluated a few PDCoV, TGEV, and PEDV singleplex PCR assays. The PDCoV PCR assays 1 and 2 cross-reacted with sparrow deltacoronaviruses 17-690-7 and 17-42824, whereas the PDCoV PCR assay 3 did not cross react with these two sparrow deltacoronaviruses and only specifically detected PDCoV. Thus, the PDCoV singleplex PCR assay 3 was selected for developing the 5-plex PCR. When two TGEV singleplex PCR assays were evaluated, the TGEV N gene-based PCR assay 1 cross-reacted with PRCV whereas the TGEV S gene-based PCR assay 2 did not cross-react with PRCV and only specifically detected TGEV. Hence, the TGEV singleplex PCR assay 2 was selected for developing the 5-plex PCR. Both the PEDV singleplex PCR assays 1 and 2 were very specific (Table 2). However, since the amplicon size of the PEDV PCR assay 1 is 198 bp and the amplicon size of the PEDV PCR assay 2 is 75 bp (Table 1), the PEDV PCR assay 2, with its smaller amplicon size, was selected to develop the 5-plex PCR.

### 3.3. Development and Validation of PEDV/PDCoV/TGEV/SADS-CoV/XIPC 5-Plex PCR for the Detection and Differentiation of Swine Enteric Coronaviruses

Based on the evaluation data obtained for singleplex PCR assays, the PEDV singleplex PCR assay 2 (probe labeled with fluorescence dye JUN), PDCoV singleplex PCR assay 3 (probe labeled with ABY), TGEV singleplex PCR assay 2 (probe labeled with VIC), and SADS-CoV singleplex PCR assay 3 (probe labeled with FAM), together with the XIPC internal positive control PCR (probe labeled with Cy5) were used to develop the PEDV/PDCoV/TGEV/SADS-CoV/XIPC 5-plex PCR, as shown in Table 1.

#### 3.3.1. Analytical Specificity of PEDV/PDCoV/TGEV/SADS-CoV/XIPC 5-Plex PCR

**Figure 1:**
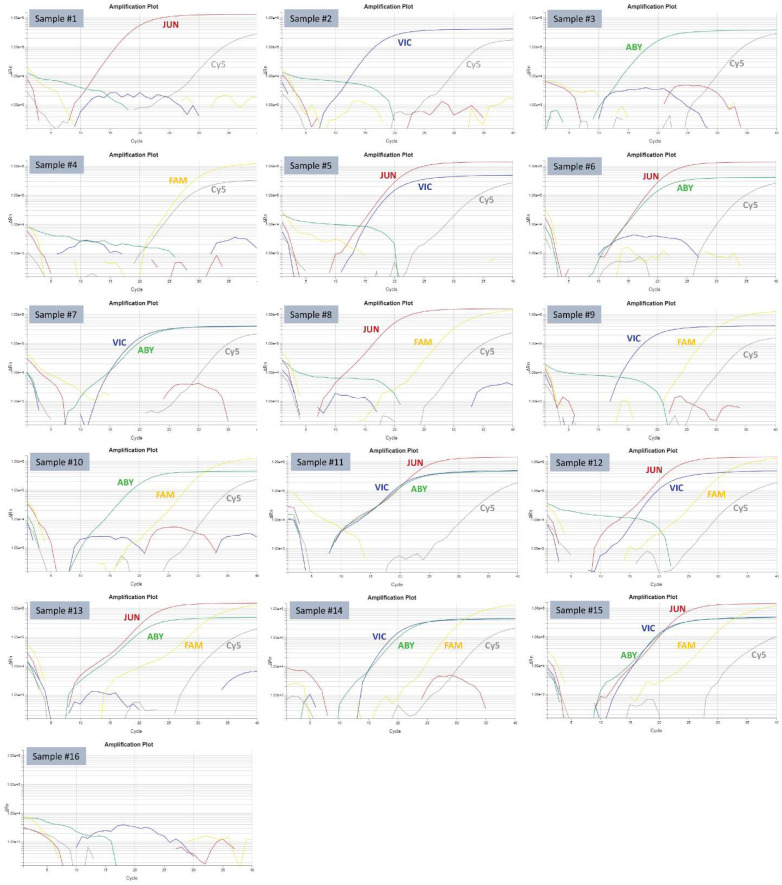
Amplification plots of 16 samples tested by the PEDV/PDCoV/TGEV/SADS-CoV/XIPC 5-plex PCR. The amplification curves corresponding to each fluorescence dye (JUN, VIC, ABY, FAM, and Cy5) are labeled accordingly. The identities of the samples #1–#16 are denoted in Table 4.

As shown in Table 2, the 5-plex PCR specifically recognized PEDV, PDCoV, TGEV, and SADS-CoV with the respective fluorescence dyes and did not cross-react with other pathogens, including the sparrow deltacoronaviruses 17-690-7 and 17-42824. When individual PEDV, TGEV, PDCoV, and SADS-CoV samples, or different manual mixtures of them at equal volume ratios, were tested by the 5-plex PCR, all the samples were correctly detected by the fluorescence dye corresponding to each virus (Table 4 and Figure 1).

#### 3.3.2. Analytical Sensitivity of PEDV/PDCoV/TGEV/SADS-CoV/XIPC 5-Plex PCR

The analytical sensitivity of the 5-plex PCR was first evaluated by testing RNA extracts from 10-fold serial dilutions of PEDV, TGEV, and PDCoV cell culture isolates in comparison with a reference PEDV/TGEV/PDCoV PCR. As shown in Table 5, the 5-plex PCR and the reference PEDV/TGEV/PDCoV PCR had similar detection endpoints for PEDV (10^−5^ dilution) with comparable Ct values between the two assays. Similarly, the two PCR assays had equivalent detection endpoints for TGEV (10^−5^ dilution) with comparable Ct values. In contrast, the 5-plex PCR assay had lower Ct values (~2 Ct differences) than the reference PCR assay in detecting PDCoV at each dilution and the 5-plex PCR had an extended detection endpoint, suggesting that the 5-plex PCR had slightly higher analytical sensitivity than the reference PCR in detecting PDCoV. The analytical sensitivity of the 5-plex PCR was further evaluated using serial dilutions of in vitro transcribed RNAs of PEDV, PDCoV, TGEV, and SADS-CoV. At higher concentrations of IVT RNAs, three replicates per dilution were tested; at lower concentrations of IVT RNAs, 20 replicates per dilution were tested. The mean Ct value, Ct range, and the percentage of PCR-positive reactions (Ct cut-off value of 37) at each dilution are summarized in Table 6. When Ct cut-off value was set at 37, the limit of detection (at least 95% of reactions are positive) of the 5-plex PCR was 8 genomic copies/reaction for PEDV, 4 genomic copies/reaction for PDCoV, 16 genomic copies/reaction for TGEV, and 6.8 genomic copies/reaction for SADS-CoV under the conditions of this study.

#### 3.3.3. Repeatability of the PEDV/PDCoV/TGEV/SADS-CoV/XIPC 5-Plex PCR

The intra-assay repeatability of the 5-plex PCR was determined by testing serial dilutions of in vitro transcribed RNAs of PEDV, PDCoV, TGEV, and SADS-CoV with three replicates in the same plate for each dilution of each virus. As shown in Table 7, the 5-plex PCR had excellent intra-assay repeatability with the average intra-assay coefficient of variation (CV) of only 0.81% for PEDV, 0.54% for PDCoV, 0.66% for TGEV and 0.80% for SADS-CoV. The inter-assay repeatability of the 5-plex PCR was determined by testing each dilution of in vitro transcribed RNAs of PEDV, PDCoV, TGEV, and SADS-CoV in three different PCR plates. The 5-plex PCR had the average inter-assay CV of 0.76% for PEDV, 1.16% for PDCoV, 0.68% for TGEV and 0.92% for SADS-CoV (Table 7), which indicated the outstanding inter-assay repeatability.

#### 3.3.4. Diagnostic Performance of PEDV/PDCoV/TGEV/SADS-CoV/XIPC 5-Plex PCR

The diagnostic performance of the 5-plex PCR was evaluated using 219 swine clinical samples (54 fecal swabs, 53 feces, 82 oral fluids, and 30 small intestines) collected from various U.S. states during 2018–2021 and compared to the reference PEDV/TGEV/PDCoV PCR. Among the 219 clinical samples, 96 samples were positive for PEDV with Ct ranges of 14.5–36, 82 samples were positive for PDCoV with Ct ranges of 14.1–36, and 12 samples were positive for TGEV with Ct ranges of 14.9–31.1, by the reference PCR with the Ct cut-off value of 36 as established at ISU VDL, indicating the wide distribution of samples from strong positive to weak positive (Table 8). Compared to the reference PCR, the diagnostic sensitivity, specificity, and agreement of the 5-plex PCR were 98.96%, 95.12%, and 96.80% for PEDV, 100%, 97.81%, and 98.63% for PDCoV, and 100%, 100%, and 100% for TGEV, respectively, when a cut-off Ct value of 37 was used for the 5-plex PCR analysis (Table 9).

Among the 219 clinical samples, there were 7 discrepant PEDV results, 3 discrepant PDCoV results, and 0 discrepant TGEV results between the 5-plex PCR and the reference PCR (Table 9). These 10 clinical samples that had the discrepant results between the 2 PCR assays all had relatively high Ct values (Table 10). To verify the identity of virus presence in these samples and to confirm the PCR results, spike gene sequencing was attempted on the 10 samples listed in Table 10. Unfortunately, due to low concentrations (high Ct values) of virus in these samples, sequencing was unsuccessful on these samples.

### 3.4. Investigation of SADS-CoV Presence by Testing Clinical Swine Samples Collected in the U.S.

In order to investigate if SADS-CoV is present in U.S. swine, 288 clinical samples archived during 2019–2020 at the ISU VDL from diarrheic pigs in the U.S. that were negative for PEDV, PDCoV, TGEV, and porcine rotavirus A, B, and C, were tested by the 5-plex PCR. All of the 288 clinical samples were negative for SADS-CoV, PEDV, PDCoV, and TGEV by the 5-plex PCR. Among the 288 samples, 68 samples were randomly selected and tested by the singleplex SADS-CoV PCR Assay 3 and all of them were negative, consistent with the 5-plex PCR result.

Subsequently, the feces, fecal swabs, and oral fluid samples submitted to the ISU VDL during March-October 2019, November-December 2020, and February-April 2021 were randomly selected for testing by the 5-plex PCR. All the tested samples were negative for SADS-CoV. Among them, 1519 samples were positive for at least one of PEDV, PDCoV, and TGEV. These 1519 samples were collected from at least 21 U.S. states and from pigs at different production stages (390 samples from adults, 321 samples from the grower/finisher stage, 316 samples from the nursery stage, 129 samples from suckling pigs, and 363 samples from pigs with unknown ages). Among the 1519 samples, the following detection frequency data were obtained: (1) 72.88% PEDV positive only, (2) 17.58% PDCoV positive only, (3) 0% TGEV positive only, (4) 8.89% PEDV and PDCoV positive, (5) 0.13% PEDV and TGEV positive, (6) 0% PDCoV and TGEV positive, and (7) 0.53% PEDV, PDCoV, and TGEV positive.

## 4. Discussion

Coronaviruses can infect a wide range of host species. The life-threatening severe acute respiratory syndrome (SARS), Middle East Respiratory Syndrome (MERS), and COVID-19 associated diseases in humans are caused by coronaviruses SARS-CoV-1, MERS-CoV, and SARS-CoV-2, respectively [45]. Coronaviruses can also infect pigs. Among the six coronaviruses infecting pigs (PEDV, PDCoV, TGEV, PRCV, PHEV, and SADS-CoV), five of them (PEDV, PDCoV, TGEV, PRCV, and PHEV) are endemic in U.S. swine [1]. Although SADS-CoV has only been reported in China, the spread of transboundary diseases between countries in recent years emphasizes the essentiality to actively monitor the potential emergence of SADS-CoV in U.S. pigs. In addition, PEDV, PDCoV, TGEV, and SADS-CoV are all enteric pathogens causing similar clinical signs, and differential diagnosis of them is needed.

In this study, we first developed and/or evaluated four SADS-CoV singleplex PCR assays. Since there are no known positive samples in the U.S. to validate SADS-CoV PCRs, validation of the SADS-CoV singleplex PCR was conducted in Huang’s lab in China where SADS-CoV positive clinical samples and SADS-CoV cell culture isolates are available [25,44]. Subsequently, one SADS-CoV singleplex PCR with the best performance together with selected PDCoV, TGEV, PEDV, and XIPC singleplex PCRs were used to develop a PEDV/PDCoV/TGEV/SADS-CoV/XIPC 5-plex TaqMan probe-based RT-rtPCR. Although numerous singleplex, duplex, and triplex PCRs have been developed to detect PEDV, PDCoV, TGEV, and SADS-CoV, so far there has been only one publication describing a PEDV/PDCoV/TGEV/SADS-CoV multiplex RT-rtPCR [42]. However, in that multiplex PCR, the TGEV primers and probe targeted the nucleocapsid gene and recognized not only TGEV but also PRCV (see Table 2 TGEV Assay 1). In contrast, in our 5-plex PCR, the TGEV primers and probe were designed to target the spike gene sequence that was absent in PRCV genome; thus, our TGEV primers and probe included in the 5-plex PCR specifically recognizes TGEV and does not cross react with PRCV (Table 2). Another disadvantage of the PEDV/PDCoV/TGEV/SADS-CoV multiplex PCR developed by Huang et al. [42] is that it does not include the primers and probe for an internal positive control. The 5-plex PCR developed in this study includes the primers and probe for an exogenous internal positive control XIPC, in addition to including primers and probes for PEDV, PDCoV, TGEV, and SADS-CoV. An exogenous internal positive control (XIPC) RNA is a target that is usually added to every reaction at the nucleic acid extraction step. The RT-rtPCR master mix contains the primers and probe for the XIPC target, so, theoretically, the XIPC should amplify in every PCR reaction. The absence of amplification of the XIPC is indicative of a problem somewhere in the process, including inhibition of the RT-rtPCR reaction. If XIPC fails and the target pathogen also fails to amplify, the result for the target pathogen is inconclusive and additional testing or resubmission of the sample is recommended. Generally speaking, a 1:1 dilution of the original clinical sample with sterile phosphate buffered saline followed by re-extraction of nucleic acids and PCR retesting could eliminate some PCR inhibitors and result in a valid XIPC PCR result. Therefore, the inclusion of XIPC in our 5-plex PCR provides an additional quality assurance approach to ensure the accuracy of the PCR results.

Initially, we attempted to develop a 6-plex PCR for use in the QuantStudio 5 thermal cycler which supports 6 channels. The plan was to label four swine enteric CoV probes with the fluorescence dyes FAM (emission wavelength ~520 nm), VIC (~550 nm), ABY (~580 nm), and JUN (~617 nm), label the internal positive control probe with the fluorescence dye Cy5.5 (~706 nm), and also include the passive reference dye Mustang Purple (~654 nm). However, calibration of the fluorescence dye Cy5.5 in the QuantStudio 5 instrument was unsuccessful, and some cross-talking signals between fluorescence dyes could not be eliminated. Due to that challenge, we redesigned the experiment to develop a 5-plex PCR including 10 primers and 5 different fluorescence dye-labeled probes. These include fluorescence dyes FAM, VIC, ABY, JUN, and Cy5 (~670 nm), to respectively label the probes for SADS-CoV, TGEV, PDCoV, PEDV, and XIPC. Since the JUN dye has similar emission spectra as the ROX dye (~610–617 nm) and the Cy5 dye has similar emission spectra as the Mustang Purple dye, a master mix containing ROX or Mustang Purple for normalization could not be used in our 5-plex PCR. Therefore, we used the TaqPath^TM^ 1-Step Multiplex Master Mix (No ROX), which does not contain a passive reference dye ROX or Mustang Purple, for our 5-plex PCR. The 5-plex PCR can be run on thermal cyclers (e.g., ABI 7500 Fast, QuantStudio 5, etc.) that support channels corresponding to FAM, VIC, ABY, JUN, and Cy5.

The 5-plex PCR has excellent analytical specificity. It did not react with other non-target pathogens, including sparrow deltacoronaviruses (Table 2). This eliminated the concerns that some previously published PDCoV assays cross-reacted with sparrow deltacoronaviruses (Table 2). Regardless of whether PEDV, PDCoV, TGEV, and SADS-CoV are present alone or in any possible combinations in a sample, the 5-plex PCR was able to correctly detect the virus identity, as demonstrated in Table 4 and Figure 1. Hence, the 5-plex PCR will be very useful to detect PEDV, PDCoV, TGEV, and SADS-CoV single infection or co-infections in clinical samples. The 5-plex PCR had comparable analytical sensitivity to the reference PEDV/TGEV/PDCoV for detecting PEDV, PDCoV, and TGEV by testing serial dilutions of the cell culture isolates (Table 5). Testing serial dilutions of PEDV, PDCoV, TGEV, and SADS-CoV IVT RNAs again confirmed that the 5-plex PCR was very sensitive and had a limit of detection of 4–16 genomic copies per reaction for the four swine enteric coronaviruses. By testing 219 clinical samples with wide Ct ranges for each of PEDV, PDCoV, and TGEV, it was demonstrated that the 5-plex PCR had comparable diagnostic performances (sensitivity, specificity, and agreement) to the reference PEDV/TGEV/PDCoV PCR. There were 7 discrepant results for PEDV between the 5-plex PCR and the reference PCR (Table 8). In fact, samples #23, #24, and #75 had PEDV Ct values of 36.4, 36.4, and 36.3 by the reference PCR and PEDV Ct values of 36.6, 35.1, and 35.4 by the 5-plex PCR (Table 9). If a Ct cut-off value of 37 was used for both PCR assays, these three samples would have had consistent PEDV results by two PCR assays. Only four samples (#27, #138, #144, and #158) had ≥3 Ct differences for PEDV by the two PCR assays. Similarly, the three samples (#113, #115, and #145) for PDCoV could have consistent results between the reference PCR and the 5-plex PCR if different Ct cut-off values were used. In this study, a Ct cut-off value of 37 was tentatively used for the 5-plex PCR. Ct cut-off value of 36 was used at ISU VDL for the reference PEDV/TGEV/PDCoV PCR (Thermo Fisher Scientific). Further optimization of the Ct cut-off values of these two PCR assays could potentially increase the equivalency results of the two PCR assays.

To investigate whether SADS-CoV is present in U.S. swine, we ran the 5-plex PCR on 288 clinical samples collected from diarrheic pigs during 2019–2020 in the U.S. that were negative for PEDV, PDCoV, TGEV, and porcine rotavirus A, B, and C. We thought that these pigs with diarrhea but testing negative for the common swine enteric viruses could be a good baseline to test for the presence of SADS-CoV in U.S. pigs. All 288 pig samples were negative for SADS-CoV, which was corroborated by testing a portion of samples using the singleplex SADS-CoV PCR Assay 3. Subsequently, we tested several thousand fecal swab, feces and oral fluid samples randomly selected from the samples submitted to the ISU VDL from 2019–2021 by the 5-plex PCR. Again, all the tested samples were negative for SADS-CoV. Interestingly, 1519 samples were found positive for at least one of PEDV, PDCoV, and TGEV. We were able to calculate the frequency of PEDV, PDCoV, and TGEV single infection or co-infection in these 1519 samples, providing some baseline data to understand the epidemiology of swine enteric coronaviruses in U.S. swine. In addition to single infection with PEDV or PDCoV in ~89% of samples, it was determined that ~10% of samples had PEDV/PDCoV co-infections and <1% of samples had PEDV/TGEV or PEDV/PDCoV/TGEV co-infections. Co-infection of swine enteric coronaviruses may cause recombination between co-infected viruses and could increase the disease severity of piglets [41,46]. Recombination occurs among CoVs [47] and two breakpoints located in the ORF1a and S genes were found to be related to CoV recombination and evolution although the exact mechanism of recombination in CoVs is still unclear [48]. A TGEV/PEDV recombinant virus (TGEV backbone but with PEDV spike gene) was identified in swine and spread across the central Eastern European countries during 2012 to 2016 [49,50,51]. A previous study also showed more severe diarrheic symptoms in the PEDV/PDCoV co-infected piglets, mainly characterized by a longer diarrheic time when compared with the single virus infection [46]. Therefore, for those samples with swine enteric CoV co-infections, additional follow-up investigations are warranted.

In addition to infecting pigs, PDCoV has been reported to cross species to infect calves [52], chicken embryos and chicken [53], and turkeys [54]. One recent article also reported that PDCoV could infect children [55], raising the public health concern of PDCoV. SADS-CoV also has broad cross-species tropism, infecting cell lines from bats, pigs, mink, nonhuman primates, humans, and other animals such as cats, dogs, hamsters, mice, rats, and chickens [37,56]. A recent paper reported that SADS-CoV replicates in primary human cells, raising concern around SADS-CoV as a potential zoonotic pathogen [57]. Most recently, young chickens are reportedly susceptible to experimental SADS-CoV infection [58]. The 5-plex PCR developed in the present study should be able to be utilized not only for testing swine samples but also for testing samples from other host species if any of these CoVs are suspected.

## 5. Conclusions

In summary, a specific and sensitive PEDV/PDCoV/TGEV/SADS-CoV/XIPC 5-plex RT-rtPCR assay was developed and thoroughly validated. This 5-plex PCR can simultaneously detect and differentiate PEDV, PDCoV, TGEV, and SADS-CoV in one PCR reaction. Although our data in the current study indicate that there was no evidence of SADS-CoV presence in the U.S. at present, the availability of the 5-plex PCR will enable us to conduct ongoing surveillance and be better prepared to respond to any future introduction.

## Figures and Tables

**Table 1:** Sequences of primers and probes used in this study.

Assay	Primer/Probe	Sequence (5′–3′)	Target	Amplicon	Reference
***Singleplex SADS-CoV PCR***					
Assay 1	SADS-N-F1	CTGACTGTTGTTGAGGTTAC	N	155 bp	Zhou L et al., J Virol Methods. 2018, 255:66–70 [35]
	SADS-N-R1	TCTGCCAAAGCTTGTTTAAC	N		
	SADS-N-Prb1	FAM/TCACAGTCT/ZEN/CGTTCTCGCAATCA/3IABkFQ	N		
Assay 2	SADS-N-F2	GCACTTTTATTACCTTGGTA	N	144 bp	Xu et al., Transbound Emerg Dis. 2019, 66:119–130 [36]
	SADS-N-R2	GTAGCAGGTTCTTTGTTAC	N		
	SADS-N-Prb2	FAM/TCCTCACGC/ZEN/AGATGCTCCTT/3IABkFQ	N		
Assay 3	SADS-N-F3	CCAGGCCTCAAAGTGGTAAAAA	N	85 bp	This study
	SADS-N-R3	TGCTTACGAGCCGGTTTAGG	N		
	SADS-N-Prb3	FAM/ACCCAAACC/ZEN/AAGAAGCAGAGCTGTCTCAC/3IABkFQ	N		
Assay 4	SADS-N-F4	TTGGCAGACTTGGGCATAGC	N	85 bp	This study
	SADS-N-R4	GTGAGACAGCTCTGCTTCTTGGT	N		
	SADS-N-Prb4	FAM/TCCAGGCCT/ZEN/CAAAGTGTAAAAATACACCC/3IABkFQ	N		
***Singleplex PDCoV PCR***					
Assay 1	PDCoV-M-RTF	CGACCACATGGCTCCAATTC	M	70 bp	Chen et al., Virology. 2015, 482:51–59 [30]
	PDCoV-M-RTR	CAGCTCTTGCCCATGTAGCTT	M		
	PDCoV-M-RTP	FAM/CACACCAGT/ZEN/CGTTAAGCATGGCAAGC/3IABkFQ	M		
Assay 2	PDCoV-N-RTF	CCTACTACTGACGCGTCTTGGTT	N	75 bp	Chen et al., Emerg Microbes Infect. 2018, 7:105 [31]
	PDCoV-N-RTR	TGCCACGAAACTGAGGATGA	N		
	PDCoV-N-RTP	VIC/TGCTCAAAGCTCAAAAC/MGB	N		
Assay 3	PDCoV-N-F2	CCAGACATGTGCCTGGTGTT	N	68 bp	This study
	PDCoV-N-R2	CCCYGCCTGAAAGTTGCT	N		
	PDCoV-N-Prb2	ABY/ARATGCTTTTCGCTGGCCACCTTG/QSY	N		
***Singleplex TGEV PCR***					
Assay 1	TGEV-N-F	TGCCATGAACAAACCAAC	N	81 bp	Huang et al., Appl Microbiol Biotechnol. 2019, 103:4943–4952 [42]
	TGEV-N-R	GGCACTTTACCATCGAAT	N		
	TGEV-N-Prb	VIC/TAGCACCACGACTACCAAGC/MGB-3′	N		
Assay 2	TGEV-S-F2	GTGGTAATATGYTRTATGGCYTACAA	S	101 bp	This study
	TGEV-S-R2	GCCAGACCATTGATTTTCAAAACT	S		
	TGEV-S-Prb2	VIC/TTGCTTATTTACATGGTGCYAGT/MGB	S		
***Singleplex PEDV PCR***					
Assay 1	PEDV-N306-F	CGCAAAGACTGAACCCACTAACCT	N	198 bp	Madson et al., Vet Microbiol. 2014, 174:60–68 [28]
	PEDV-N503-R	TTGCCTCTGTTGTTACTTGGAGAT	N		
	PEDV-N469-Prb	FAM/TGTTGCCATTACCACGACTCCTGC/QSY	N		
Assay 2	PEDV-N1195-F	GAAGAGGCCATCTACGATGATGT	N	75 bp	This study
	PEDV-N1269-R	AACAGCTGTGTCCCATTCCAA	N		
	PEDV-N1221-Prb	JUN/TGTGCCATCTGATGTGACTCATGCCA/QSY	N		
***PEDV/PDCoV/TGEV/SADS-CoV/XIPC 5-plex PCR***					
	PEDV-N1195-F	GAAGAGGCCATCTACGATGATGT	N	75 bp	This study
	PEDV-N1269-R	AACAGCTGTGTCCCATTCCAA	N		
	PEDV-N1221-Prb	**JUN**/TGTGCCATCTGATGTGACTCATGCCA/**QSY**	N		
	PDCoV-N-F2	CCAGACATGTGCCTGGTGTT	N	68 bp	This study
	PDCoV-N-R2	CCCYGCCTGAAAGTTGCT	N		
	PDCoV-N-Prb2	**ABY**/ARATGCTTTTCGCTGGCCACCTTG/**QSY**	N		
	TGEV-S-F2	GTGGTAATATGYTRTATGGCYTACAA	S	101 bp	This study
	TGEV-S-R2	GCCAGACCATTGATTTTCAAAACT	S		
	TGEV-S-Prb2	**VIC**/TTGCTTATTTACATGGTGCYAGT/**MGB**	S		
	SADS-N-F3	CCAGGCCTCAAAGTGGTAAAAA	N	85 bp	This study
	SADS-N-R3	TGCTTACGAGCCGGTTTAGG	N		
	SADS-N-Prb3	**FAM**/ACCCAAACC/ZEN/AAGAAGCAGAGCTGTCTCAC/**QSY**	N		

Note: The singleplex PCR assays used to develop the 5-plex PCR are shown as highlighted.

**Table 2:** Analytical specificity of various singleplex and multiplex PCRs evaluated in this study.

Pathogens	SADS-CoV Singleplex Assay				PDCoV Singleplex Assay			TGEV Singleplex Assay		PEDV Singleplex Assay		5-Plex PCR	Reference PEDV/
	Assay 1	Assay 2	Assay 3 *	Assay 4	Assay 1	Assay 2	Assay 3 *	Assay 1	Assay 2 *	Assay 1	Assay 2 *		TGEV/PDCoV PCR
PEDV	≥40	≥40	≥40	≥40	≥40	≥40	≥40	≥40	≥40	18.21	17.05	16.09	15.51
TGEV Purdue	≥40	≥40	≥40	≥40	≥40	≥40	≥40	15.00	17.43	≥40	≥40	15.87	15.41
TGEV Miller	≥40	≥40	≥40	≥40	≥40	≥40	≥40	17.23	19.51	≥40	≥40	17.53	16.93
PDCoV	≥40	≥40	≥40	≥40	17.93	17.45	14.01	≥40	≥40	≥40	≥40	16.52	17.35
SADS-CoV RNA	28.35	26.05	25.85	25.15	≥40	≥40	≥40	≥40	≥40	≥40	≥40	27.56	≥40
PRCV	≥40	≥40	≥40	≥40	≥40	≥40	≥40	18.04	≥40	≥40	≥40	≥40	≥40
PHEV	≥40	≥40	≥40	≥40	≥40	≥40	≥40	≥40	≥40	≥40	≥40	≥40	≥40
Sparrow DCoV (17-690-7)	≥40	≥40	≥40	≥40	34.72	30.91	≥40	≥40	≥40	≥40	≥40	≥40	≥40
Sparrow DCoV (17-42824)	≥40	≥40	≥40	≥40	33.08	27.30	≥40	≥40	≥40	≥40	≥40	≥40	≥40
Porcine rotavirus (A, B, C)	≥40	≥40	≥40	≥40	≥40	≥40	≥40	≥40	≥40	≥40	≥40	≥40	≥40
PCV 2	≥40	≥40	≥40	≥40	≥40	≥40	≥40	≥40	≥40	≥40	≥40	≥40	≥40
Influenza A virus	≥40	≥40	≥40	≥40	≥40	≥40	≥40	≥40	≥40	≥40	≥40	≥40	≥40
PPIV-1	≥40	≥40	≥40	≥40	≥40	≥40	≥40	≥40	≥40	≥40	≥40	≥40	≥40
Pseudorabies virus	≥40	≥40	≥40	≥40	≥40	≥40	≥40	≥40	≥40	≥40	≥40	≥40	≥40
Senecavirus A	≥40	≥40	≥40	≥40	≥40	≥40	≥40	≥40	≥40	≥40	≥40	≥40	≥40
PRRSV-1 Lelystad	≥40	≥40	≥40	≥40	≥40	≥40	≥40	≥40	≥40	≥40	≥40	≥40	≥40
PRRSV-2 VR-2385	≥40	≥40	≥40	≥40	≥40	≥40	≥40	≥40	≥40	≥40	≥40	≥40	≥40
Bacteria ^‡^	≥40	≥40	≥40	≥40	≥40	≥40	≥40	≥40	≥40	≥40	≥40	≥40	≥40

* Singleplex PCR assays that were selected to develop the PEDV/PDCoV/TGEV/SADS-CoV/XIPC 5-plex PCR. ‡ Bacterial pathogens for evaluating assay specificity include *E. coli*, *Salmonella typhimurium*, *Clostridium difficile*, *Clostridium perfringens*, *Brachyspira hyodysenteriae*, *Mycoplasma hyopneumoniae*, *Mycoplasma hyorhinis*, *Mycoplasma hyosynoviae*, *Actinobacillus pleuropneumonia*, *Actinobacillus suis*, *Streptococcus suis*, *Glaesserella (Haemophilus) parasuis*, *Bordetella bronchiseptica*, *Pasteurella multocida*, and *Trueperella pyogenes*.

**Table 3:** Analytical sensitivity of Singleplex SADS-CoV PCR assays 2 and 3 by testing serial dilutions of a SADS-CoV cell culture isolate *.

Dilution	Theoretical Titer (TCID_50_/mL)	SADS-CoV ASSAY 2			SADS-CoV ASSAY 3		
		Result 1	Result 2	Result 3	Result 1	Result 2	Result 3
10^−2^	5 × 10^5^	20.64	20.68	20.69	20.76	20.76	20.69
10^−3^	5 × 10^4^	24.05	24.03	24.03	24.01	23.97	24.13
10^−4^	5 × 10^3^	27.51	27.47	27.59	27.44	27.36	27.42
10^−5^	5 × 10^2^	31.00	30.99	31.23	31.10	31.06	31.24
10^−6^	5 × 10^1^	34.37	34.25	33.98	34.78	34.10	34.95
10^−7^	5 × 10^0^	38.45	37.24	38.23	37.45	37.52	36.58
10^−8^	5 × 10^−1^	≥40	≥40	≥40	37.26	37.15	≥40
10^−9^	5 × 10^−2^	≥40	≥40	≥40	≥40	≥40	≥40

* The work was conducted in Huang’s lab in China.

**Table 4:** Analytical specificity of the 5-plex PCR examined by mixtures of different viruses.

Sample No.	Sample Detail (Volume Mixing Ratio)	PEDV/PDCoV/TGEV/SADS-CoV/XIPC 5-Plex PCR Ct Value				
		PEDV (JUN)	TGEV (VIC)	PDCoV (ABY)	SADS-CoV (FAM)	XIPC (Cy5)
Sample #1	PEDV USA/IN19338/2013	15.208	≥40	≥40	≥40	31.359
Sample #2	TGEV Purdue (VR-763)	≥40	13.874	≥40	≥40	31.658
Sample #3	PDCoV USA/IL/2014	≥40	≥40	16.55	≥40	31.891
Sample #4	SADS-CoV N-gene IVT RNA	≥40	≥40	≥40	26.057	25.991
Sample #5	PEDV & TGEV (1:1)	17.245	16.413	≥40	≥40	31.079
Sample #6	PEDV & PDCoV (1:1)	17.375	≥40	17.332	≥40	32.060
Sample #7	TGEV & PDCoV (1:1)	≥40	15.993	17.834	≥40	32.358
Sample #8	PEDV & SADS-CoV (1:1)	15.646	≥40	≥40	26.771	32.073
Sample #9	TGEV & SADS-CoV (1:1)	≥40	15.076	≥40	26.870	32.533
Sample #10	PDCoV & SADS-CoV (1:1)	≥40	≥40	16.936	27.144	32.941
Sample #11	PEDV & TGEV & PDCoV (1:1:1)	18.434	15.574	17.216	≥40	33.345
Sample #12	PEDV & TGEV & SADS-CoV (1:1:1)	17.539	16.991	≥40	26.980	32.119
Sample #13	PEDV & PDCoV & SADS-CoV (1:1:1)	17.215	≥40	17.22	27.434	32.812
Sample #14	TGEV & PDCoV & SADS-CoV (1:1:1)	≥40	16.454	18.261	27.469	32.630
Sample #15	PEDV & TGEV & PDCoV & SADS-CoV (1:1:1:1)	19.124	16.782	17.869	28.350	35.304
Sample #16	Nuclease-free water without XIPC	≥40	≥40	≥40	≥40	≥40

Notes: For samples #5–#15, the respective viruses were manually mixed at the equal volume ratio; the PEDV probe was labeled with fluorescence dye JUN; the TGEV probe was labeled with fluorescence dye VIC; the PDCoV probe was labeled with fluorescence dye ABY; the SADS-CoV probe was labeled with fluorescence dye FAM; XIPC is an internal positive control and its probe was labeled with fluorescence dye CY5.

**Table 5:** Analytical sensitivity of PEDV/PDCoV/TGEV/SADS-CoV/XIPC 5-plex PCR and the reference PEDV/TGEV/PDCoV PCR by testing serial dilutions of virus isolates.

Dilution	PEDV Isolate IN19338/2013		TGEV Purdue Isolate (VR-763)		PDCoV Isolate (USA/IL/2014)	
	5-Plex PCR Ct Value	Reference PCR Ct Value	5-Plex PCR Ct Value	Reference PCR Ct Value	5-Plex PCR Ct Value	Reference PCR Ct Value
10^−1^	21.09	21.74	21.24	21.32	20.19	21.98
10^−1^	20.78	21.74	21.35	21.52	20.19	22.27
10^−1^	20.79	21.75	21.22	21.49	20.29	21.79
10^−2^	24.41	25.12	25.05	25.22	23.99	25.96
10^−2^	24.55	25.16	25.02	24.83	23.86	26.05
10^−2^	24.01	25.27	25.08	25.16	23.98	25.96
10^−3^	27.85	28.71	28.57	29.32	27.45	29.65
10^−3^	27.32	29.01	28.98	29.24	27.02	29.45
10^−3^	27.09	28.88	28.71	29.01	26.98	29.62
10^−4^	30.50	32.05	31.99	33.05	30.97	32.99
10^−4^	30.31	32.09	32.34	32.44	30.38	32.73
10^−4^	29.58	32.01	32.41	32.35	30.15	32.65
10^−5^	35.86	35.92	35.15	35.68	34.19	35.51
10^−5^	36.29	37.51	36.06	35.09	33.88	35.89
10^−5^	35.79	36.48	35.45	36.32	32.96	36.96
10^−6^	≥40	38.56	≥40	38.54	37.89	≥40
10^−6^	≥40	38.57	37.29	38.27	36.57	≥40
10^−6^	36.59	39.56	≥40	38.53	37.21	39.38
10^−7^	≥40	≥40	≥40	≥40	38.51	≥40
10^−7^	≥40	39.61	38.63	≥40	≥40	39.11
10^−7^	≥40	≥40	≥40	≥40	37.57	≥40

**Table 6:** Limit of detection of PEDV/PDCoV/TGEV/SADS-CoV/XIPC 5-plex PCR by testing serial dilutions of IVT RNAs.

Genomic Copies per Reaction	PEDV/PDCoV/TGEV/SADS-CoV/XIPC 5-Plex PCR *	
*PEDV N-gene IVT RNA*	% (No. of Pos for PEDV)	Mean Ct (range)
8 × 10^3^	100% (3/3)	24.28 (24.24–24.33)
8 × 10^2^	100% (3/3)	27.71 (27.63–27.79)
8 × 10^1^	100% (20/20)	31.35 (30.85–32.26)
16	100% (20/20)	33.76 (32.72–35.04)
8	100% (20/20)	34.55 (33.36–35.80)
4	80% (16/20)	35.7 (34.31–38.05)
2	45% (9/20)	37.53 (35.27–40)
1	5% (1/20)	38.70 (36.85–40)
*PDCoV N-gene IVT RNA*	% (No. of Pos for PDCoV)	Mean Ct (range)
8 × 10^3^	100% (3/3)	24.02 (23.96–24.11)
8 × 10^2^	100% (3/3)	27.64 (27.56–27.72)
8 × 10^1^	100% (3/3)	31.41 (31.15–31.64)
16	100% (20/20)	33.95 (33.40–34.62)
8	100% (20/20)	34.93 (34.09–36.46)
4	95% (19/20)	36.24 (34.59–37.77)
2	15% (3/20)	37.92 (36.61–40)
1	0% (0/20)	38.71 (36.95–40)
*TGEV S-gene IVT RNA*	% (No. of Pos for TGEV)	Mean Ct (range)
8 × 10^3^	100% (3/3)	25.28 (25.21–25.34)
8 × 10^2^	100% (3/3)	28.81 (28.75–28.85)
8 × 10^1^	100% (3/3)	32.71 (32.67–32.77)
16	95% (19/20)	35.75 (34.83–37.34)
8	25% (5/20)	37.47 (35.87–38.75)
4	0% (0/20)	39.16 (37.14–40)
2	0% (0/20)	39.79 (37.95–40)
1	0% (0/20)	39.96 (39.61–40)
*SADS-CoV N-gene IVT RNA*	% (No. of Pos for SADS-CoV)	Mean Ct (range)
1.36 × 10^4^	100% (3/3)	23.87 (23.84–23.90)
1.36 × 10^3^	100% (3/3)	27.27 (27.15–27.35)
1.36 × 10^2^	100% (3/3)	30.65 (30.54–30.75)
27.2	100% (20/20)	32.91 (32.35–34.58)
13.6	100% (20/20)	34.23 (33.54–35.21)
6.8	100% (20/20)	35.41 (34.53–36.76)
3.4	85% (17/20)	36.38 (35.25–38.55)
1.7	35% (7/20)	37.93 (35.94–40)

* The PEDV/PDCoV/TGEV/SADS-CoV/XIPC 5-plex PCR Ct < 37 was considered positive for each virus.

**Table 7:** Repeatability of PEDV/PDCoV/TGEV/SADS-CoV/XIPC 5-plex PCR by testing serial dilutions of in vitro transcribed RNA.

*PEDV N-Gene IVT RNA* *(Genomic Copies per Reaction)*	Intra-Assay (3 Replicates for Each Dilution)		Inter-Assay (3 Plates for Each Dilution)	
	Mean Ct Value	CV, %	Mean Ct Value	CV, %
8.0 × 10^6^	14.82	1.05	14.90	0.68
8.0 × 10^5^	18.22	0.81	18.21	0.15
8.0 × 10^4^	21.28	0.83	21.16	0.76
8.0 × 10^3^	24.87	0.80	24.88	0.85
8.0 × 10^2^	28.12	0.42	28.31	1.35
8.0 × 10^1^	31.50	0.35	31.51	0.78
8.0 × 10^0^	35.93	1.38	35.73	0.78
	Average intra-assay CV, %	0.81	Average inter-assay CV, %	0.76
*PDCoV N-Gene IVT RNA* *(Genomic Copies per Reaction)*	Intra-Assay (3 Replicates for Each Dilution)		Inter-Assay (3 plates for Each Dilution)	
	Mean Ct Value	CV, %	Mean Ct Value	CV, %
8.0 × 10^6^	14.82	0.49	15.18	2.09
8.0 × 10^5^	18.24	0.16	18.53	1.45
8.0 × 10^4^	22.31	0.13	22.64	1.28
8.0 × 10^3^	24.58	0.68	24.65	0.26
8.0 × 10^2^	28.52	0.55	28.69	0.51
8.0 × 10^1^	32.67	0.24	33.11	1.16
8.0 × 10^0^	35.90	1.56	36.41	1.37
	Average intra-assay CV, %	0.54	Average inter-assay CV, %	1.16
*TGEV S-Gene IVT RNA* *(Genomic Copies per Reaction)*	Intra-Assay (3 Replicates for Each Dilution)		Inter-assay (3 plates for each dilution)	
	Mean Ct Value	CV, %	Mean Ct Value	CV, %
8.0 × 10^6^	15.39	0.10	15.43	0.23
8.0 × 10^5^	18.92	0.36	19.09	0.78
8.0 × 10^4^	22.92	0.22	23.01	0.37
8.0 × 10^3^	25.57	0.16	25.59	0.27
8.0 × 10^2^	29.50	0.09	29.85	1.13
8.0 × 10^1^	33.51	0.67	33.77	0.86
8.0 × 10^0^	37.90	3.01	37.67	1.13
	Average intra-assay CV, %	0.66	Average inter-assay CV, %	0.68
*SADS-CoV N-Gene IVT RNA (Genomic Copies per Reaction)*	Intra-Assay (3 Replicates for Each Dilution)		Inter-Assay (3 Plates for Each Dilution)	
	Mean Ct Value	CV, %	Mean Ct Value	CV, %
1.36 × 10^7^	14.50	0.39	14.49	0.13
1.36 × 10^6^	17.81	1.10	17.57	1.37
1.36 × 10^5^	21.77	0.35	21.43	1.41
1.36 × 10^4^	24.76	0.69	24.59	0.68
1.36 × 10^3^	28.57	0.89	28.31	0.88
1.36 × 10^2^	31.92	0.83	32.06	1.09
1.36 × 10^1^	35.30	1.32	35.54	0.90
	Average intra-assay CV, %	0.80	Average inter-assay CV, %	0.92

**Table 8:** Specimen types of 219 clinical samples and Ct ranges of positive samples tested by the reference PEDV/PDCoV/TGEV PCR.

Specimen Type	Number	Reference PCR—PEDV Positive (Ct < 36)		Reference PCR—PDCoV Positive (Ct < 36)		Reference PCR—TGEV Positive (Ct < 36)	
		Number	Ct Range	Number	Ct range	Number	Ct range
Fecal swab	54	21	16.6–33.2	13	15.5–35.7	3	25.4–31.1
Feces	53	20	14.5–30.2	32	14.1–36	9	14.9–28.4
Oral fluid	82	33	17.8–36	36	17.8–36	0	
Small intestine	30	22	15.2–35.2	1	20.7	0	
Total	219	96		82		12	

**Table 9:** Diagnostic performance of PEDV/PDCoV/TGEV/SADS-CoV/XIPC 5-plex PCR in comparison with the reference PEDV/TGEV/PDCoV PCR on clinical samples *.

		Reference PCR—PEDV		
		Positive	Negative	Total
5-plex PCR—PEDV	Positive	95	6	101
	Negative	1	117	118
	Total	96	123	219
Sensitivity 98.96%; specificity 95.12%; agreement 96.80%				
		**Reference PCR—PDCoV**		
		Positive	Negative	Total
5-plex PCR—PDCoV	Positive	82	3	85
	Negative	0	134	134
	Total	82	137	219
Sensitivity 100%; specificity 97.81%; agreement 98.63%				
		**Reference PCR—TGEV**		
		Positive	Negative	Total
5-plex PCR—TGEV	Positive	12	0	12
	Negative	0	207	207
	Total	12	207	219
Sensitivity 100%; specificity 100%; agreement 100%				

* Ct < 37 was considered positive for the PEDV/PDCoV/TGEV/SADS-CoV/XIPC 5-plex and Ct < 36 was considered positive for the reference PEDV/TGEV/PDCoV PCR.

**Table 10:** Discrepancies on clinical samples between the PEDV/PDCoV/TGEV/SADS-CoV/XIPC 5-plex PCR and the reference PEDV/TGEV/PDCoV PCR *.

Sample ID	Specimen	Reference PCR			5-Plex PCR			
		PEDV	PDCoV	TGEV	PEDV	PDCoV	TGEV	SADS-CoV
Sample_#23	Fecal swab	36.4	≥40	≥40	36.6	≥40	≥40	≥40
Sample_#24	Fecal swab	36.4	≥40	≥40	35.1	≥40	≥40	≥40
Sample_#27	Fecal swab	39.4	≥40	≥40	36.4	≥40	≥40	≥40
Sample_#75	Feces	36.3	34.6	≥40	35.4	31.4	≥40	≥40
Sample_#138	Oral fluid	35.0	≥40	≥40	≥40	≥40	≥40	≥40
Sample_#144	Oral fluid	38.5	≥40	≥40	35.6	≥40	≥40	≥40
Sample_#154	Oral fluid	≥40	28.6	≥40	35.8	27.4	≥40	≥40
Sample_#113	Oral fluid	21.5	37.0	≥40	20.2	33.5	≥40	≥40
Sample_#115	Oral fluid	24.6	36.8	≥40	23.8	36.4	≥40	≥40
Sample_#145	Oral fluid	39.0	37.7	≥40	39.2	36.0	≥40	≥40

* Ct < 37 was considered positive for the PEDV/PDCoV/TGEV/SADS-CoV/XIPC 5-plex and Ct < 36 was considered positive for the reference PEDV/TGEV/PDCoV PCR.

## Data Availability

All of the data have been provided in this manuscript.
